# Comparative Analysis of the Transcriptome of Latent Autoimmune Diabetes in Adult (LADA) Patients from Eastern China

**DOI:** 10.1155/2019/8616373

**Published:** 2019-12-14

**Authors:** Yuqiao Ji, Dongmei Jiang, Jian Liu, Xiaolong Chen, Tian Xia, Zhujun Yin, Lei Li, Hao Jin, Hongmei Chen, Mingzhong Sun

**Affiliations:** ^1^Department of Clinical Laboratory, Affiliated Yancheng Hospital, School of Medicine, Southeast University, Yancheng, Jiangsu 224001, China; ^2^Reproductive & Developmental Biology Laboratory, National Institute of Environmental Health Sciences (NIEHS), Research Triangle Park, NC 27709, USA; ^3^East China Normal University, 500 Dongchuan Road, Shanghai 200241, China

## Abstract

Latent autoimmune diabetes in adults (LADA) is characterized as a slow-progressing form of autoimmune diabetes. LADA resembles some phenotypes of type 1 diabetes (T1D) and type 2 diabetes (T2D), frequently leading to misdiagnosis and inappropriate therapeutic strategies. Understanding its transcriptome profiles aids in revealing the detailed molecular mechanisms of LADA and its therapy. In the present study, we performed RNA-seq analysis of LADA patients from Eastern China and showed that LADA exhibited 277 differentially expressed genes (DEGs) with 199 upregulated and 78 downregulated. Gene ontology and KEGG pathway enrichment analysis revealed that these DEGs were mainly related to immune function and cell death and growth. Furthermore, a comparison of DEGs in LADA with those in T1D and T2D identified from the online databases showed that there are very few overlapped genes between LADA and T1D or T2D, confirming LADA to be a distinct type of diabetes from T1D or T2D. In summary, our comprehensive analysis may aid in the understanding and treatment of LADA patients in Eastern China.

## 1. Introduction

Diabetes, a metabolic disorder characterized by hyperglycemia, belongs to one of the top diseases causing disability in China and a huge health burden in China [1]. And it is the seventh leading cause of death in the United States, which has been recently shown to be far underreported [2, 3]. There are 2 main categories of diabetes, type 1 (T1D) and type 2 (T2D) with the latter accounting for more than 90% of all cases. T1D, which usually begins in childhood, is caused by immune-mediated absolute insulin deficiency, while T2D is caused by insulin resistance [4, 5]. Among all diabetes, around 10% of them are diagnosed as latent autoimmune diabetes in adults (LADA) [6]. These patients are usually over age 30 and had the presence of diabetes-associated autoantibodies (*e.g.*, glutamic acid decarboxylase (GAD65 Ab)) [6]. LADA also shares genetic features with both T1D and T2D [6]. Meanwhile, LADA patients are often misdiagnosed as T2D due to the similar phenotypes while it has worse hemoglobin A1c (HbA1c) levels than T2D [6, 7]. Therefore, LADA, resembling some of the phenotypes of T1D and T2D, is often defined as type 1.5 diabetes.

Diabetes is a complex set of multifactorial diseases involving genetic, environmental, and lifestyle factors. Great efforts have been made to study the genetic basis of LADA in relation to T1D and T2D based on the overlap in the pathological features between LADA and these two forms of diabetes. The high-risk T1D gene variants of both protein tyrosine phosphatase non-receptor 22 (*PTPN22*) and insulin (*INS*) are also associated with LADA [8]. Similarly, a T2D gene variant of transcription factor 7-like 2 (*TCF7L2*) has been found to play important roles in LADA by disrupting *β*-cell function and development [9]. However, there is a limited systemic analysis of the gene expression profile between LADA and T1D or T2D.

In the present study, we identified the DEGs in LADA patients by performing RNA-seq analysis of the transcriptome profiles between LADA and healthy controls, followed by RT-PCR validation of DEGs. We then did the pathway analysis of these DEGs in LADA patients. Overlapping analyses were further conducted to analyze the common genes between LADA and T1D or T2D patients. Our study provides a genome-wide scale to dissect the gene expression profiles among these three subtypes of diabetes.

## 2. Materials and Methods

### 2.1. Subjects

The study protocol was approved by the Fourth People's Hospital of Yancheng, and written informed consent was obtained. The inclusion and exclusion criteria of patients and controls here were diagnosed based on the rules suggested by the Chinese Diabetes Society. And these patients do not have other severe diseases than diabetes, which can help us reduce the potential effects of other diseases on our transcriptome analysis.

### 2.2. RNA Extraction and RNA-seq

Peripheral blood mononuclear cells (PBMCs) were separated from collected whole blood using density gradient centrifugation. Total RNA was extracted from PBMCs using a RNeasy kit (Qiagen, Valencia, CA) and quantified with NanoDrop 1000 (Thermo Fisher Scientific, Waltham, MA). The cDNA sequencing libraries were prepared using Illumina's TruSeq Sample Preparation Kit (San Diego, CA) and the sequencing was performed using the Illumina Genome Analyzer. RNA-seq data were analyzed using R with various packages. The sequencing reads were mapped to the human genome (hg19) using the TopHat package and annotated with a GTF file. Oebiotech company finished the sequence. The differential analysis of genes was conducted on counts using the DESeq2 package. Differentially expressed genes (DEGs) were identified as such if the fold change > 2 and the *p* value < 0.05. Gene ontology (GO) enrichment and enriched KEGG (Kyoto Encyclopedia of Genes and Genomes) pathways were performed. All the raw data of RNA-seq were deposited into GEO databases (GSE136053).

### 2.3. Real-Time qRT-PCR

Total RNA extracted from PBMCs was reverse transcribed using the SuperScript III First-Strand Synthesis System (Thermo Fisher Scientific, Waltham, MA) according to the manufacturer's instructions. Synthesized cDNA was used to quantify the gene expression on the Applied Biosystems 7500 Real-Time PCR system (Applied Biosystems, Foster City, CA) using the SYBR-green PCR master mix (Thermo Fisher Scientific, Waltham, MA). The relative mRNA expression was calculated in comparison to the control of GAPDH using the equation 2^-*ΔΔ*Ct^.

### 2.4. Data Analysis

Data were represented as mean ± SEM. Statistical analysis was performed with GraphPad Prism 6.0 (Graphpad Software Inc., San Diego, CA). Comparisons between the two groups were performed using Student's *t*-test. A *p* value < 0.05 was considered significant.

## 3. Results

### 3.1. Identification of DEGs in LADA in Comparison with Healthy Controls

**Figure 1:**
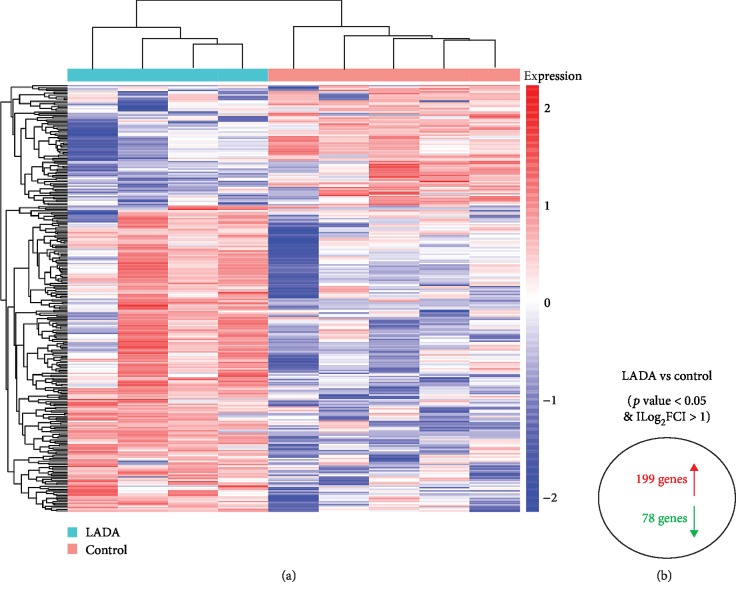
Identification of DEGs in the blood of LADA using RNA-seq. (a) The hierarchical clustering of the log_2_ fold change expression values of DEGs in LADA. (b) The summary of upregulated and downregulated genes in LADA compared with healthy controls.

To study the gene expression profiles in LADA, we performed RNA-seq analysis of LADA patients and healthy controls. In the blood of LADA patients, we identified 277 DEGs, among which 199 genes were upregulated and 78 genes were downregulated (Supplementary [Supplementary-material supplementary-material-1]). Hierarchical clustering heat map of the DEGs revealed that 4 LADA patients were clustered together and were distinctly separated from the clustering of 5 healthy controls (Figure 1). These data demonstrated that LADA exhibited unique gene expression profiles in comparison with that of healthy controls.

### 3.2. Gene Ontology Analysis of DEGs in LADA

**Figure 2:**
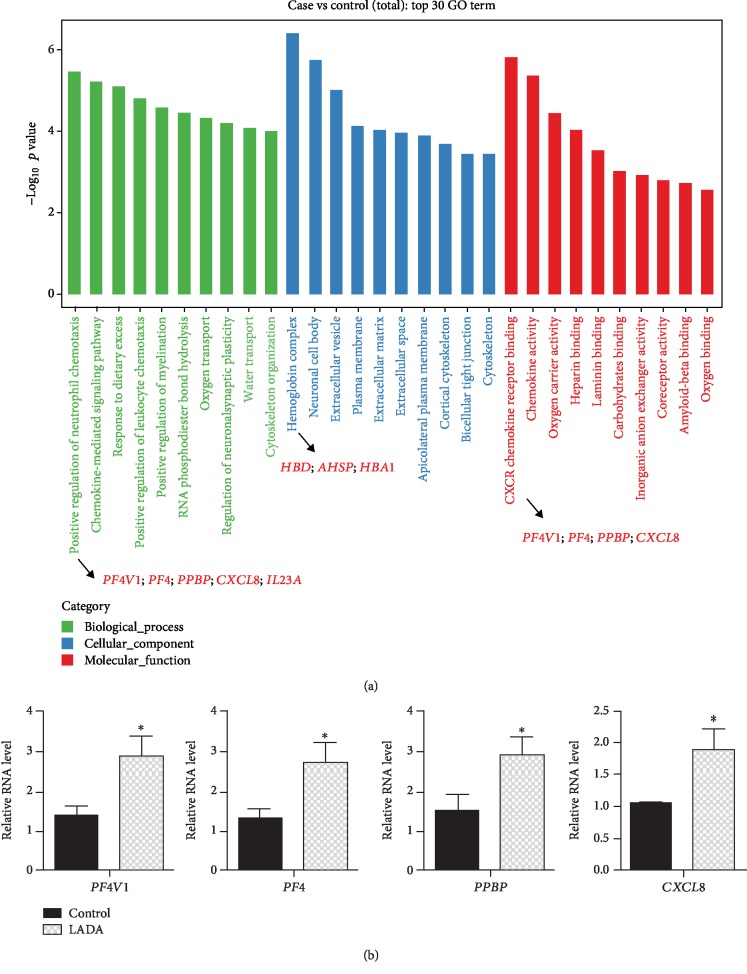
GO analysis of differentially expressed genes. (a) The bar chart shows the top 30 GO terms enriched in DEGs in LADA. These GO terms fell into 3 categories: biological process, cellular component, and molecular function. (b) Bar charts show the validation of DEGs using RT-PCR. ^∗^*p* < 0.05; Student's *t*-test, *n* = 3‐5 in each group.

To further characterize the functions of these DEGs, we performed gene ontology analysis. As shown in Figure 2(a), the top 30 GO terms were significantly enriched in 3 cellular function categories, namely biological process, cellular component, and molecular function. Importantly, many of these enriched GO terms are closely related to immune function, such as positive regulation of neutrophil chemotaxis (GO:0090023), chemokine-mediated signaling pathway (GO:0070098), and CXCR chemokine receptor binding (GO:0045236) (Supplementary [Supplementary-material supplementary-material-1]). Next, we validated the gene expression of several key genes in the top GO terms of both biological process and molecular function categories. The real-time RT-PCR experiment showed that *PF4V1*, *PF4*, *PPBP*, and *CXCL8* were significantly increased in the LADA patients compared with the healthy controls (Figure 2(b)).

### 3.3. KEGG Pathway Analysis of the Transcriptome Profiles of LADA

**Figure 3:**
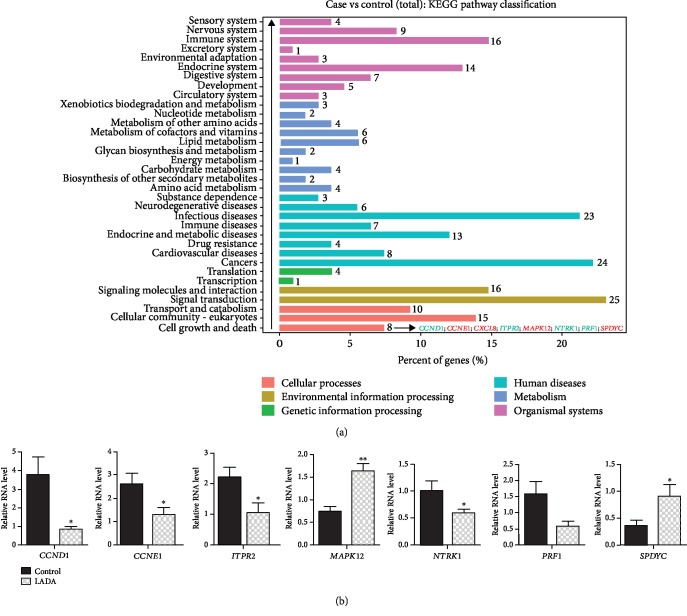
KEGG pathway classification analysis of DEGs. (a) The bar chart shows the classification of enriched GO terms into 6 classifications in LADA. The numbers beside each bar represent the amount of DEGs in each GO term. (b) Bar charts show the validation of DEGs using RT-PCR. ^∗^*p* < 0.05, ^∗∗^*p* < 0.01; Student's *t*-test, *n* = 3‐5 in each group.

**Figure 4:**
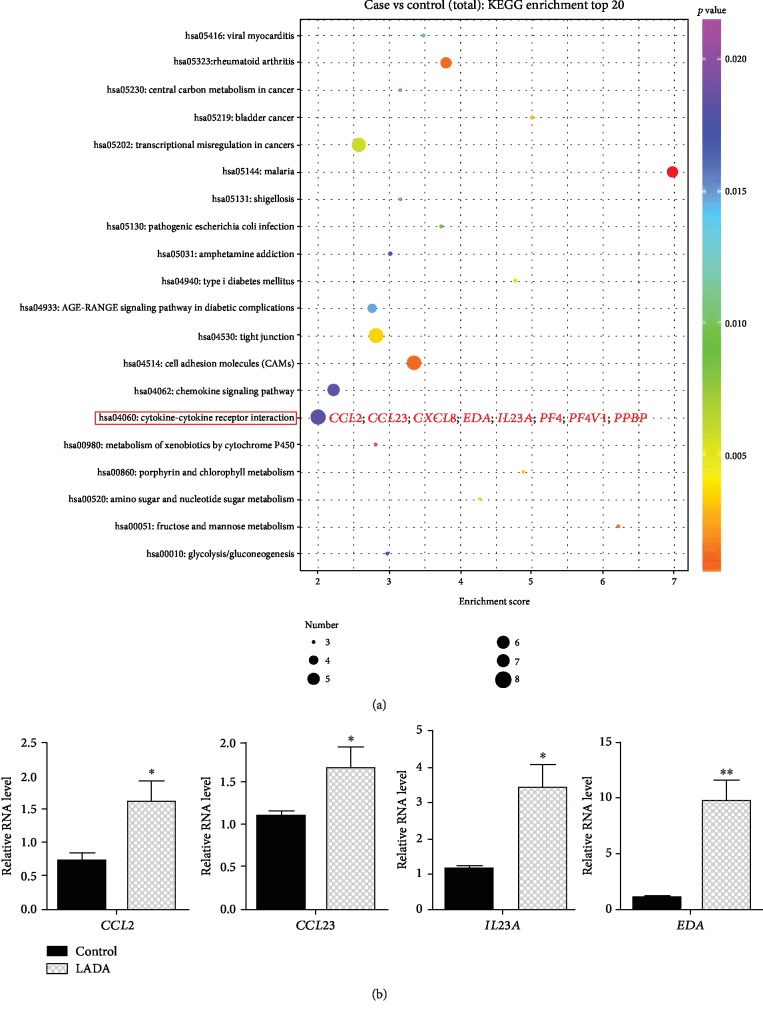
KEGG pathway analysis of DEGs. (a) The advanced bubble chart shows the enrichment of DEGs in signaling pathways. The *y*-axis represents signaling pathways. The *x*-axis represents an enrichment score. Symbol size and color represent the amount of DEGs and *p* value in each pathway, respectively. (b) Bar charts show the validation of DEGs using RT-PCR. ^∗^*p* < 0.05, ^∗∗^*p* < 0.01; Student's *t*-test, *n* = 3‐5 in each group.

To understand the molecular mechanisms of these DEGs in the pathophysiology of LADA, we performed KEGG pathway analysis. From the KEGG pathway classification analysis, we found that cell death and growth ranked first in the KEGG pathway among all, indicating that these DEGs play important roles in regulating cell survival (Figure 3(a) and Supplementary [Supplementary-material supplementary-material-1]). We validated the expression of 8 genes in this pathway using real-time qRT-PCR and demonstrated that *CCND1*, *CCNE1*, *ITPR2*, *NTRK1*, and *PRF1* were decreased in LADA, while *MAPK12* and *SPDYC* were increased in LADA compared with the healthy controls (Figure 3(b)). Furthermore, cytokine-cytokine receptor interaction pathway has the most number of DEGs (Figure 4(a) and Supplementary [Supplementary-material supplementary-material-1]), indicating that this pathway plays important roles in the development of LADA. The upregulation of representative DEGs in this pathway was further confirmed by RT-PCR (Figure 4(b)). These results revealed the autoimmune feature of LADA.

### 3.4. Overlapping Analysis of DEGs between LADA and T1D or T2D

**Figure 5:**
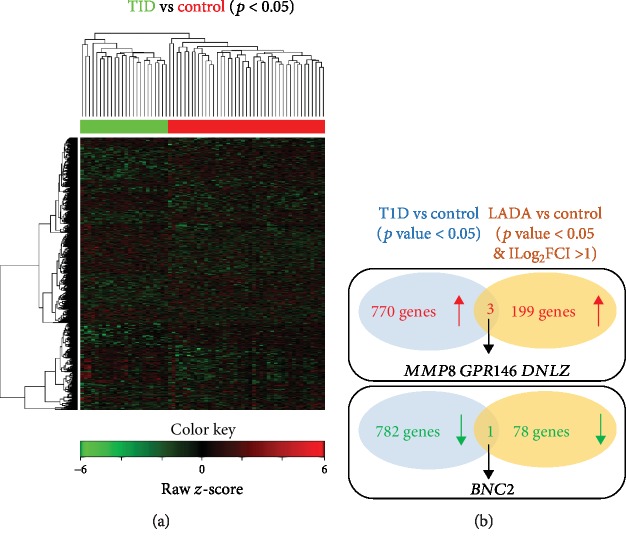
Overlapping analysis of DEGs between LADA and T1D. (a) The heat map shows the hierarchical clustering of the DEGs in T1D (solid green bar) and control (solid red bar) groups. Expression values in each row are *z*-score normalized. (b) Venn diagrams show the overlap of commonly upregulated or downregulated genes in T1D and LADA. The identified common genes are indicated.

**Figure 6:**
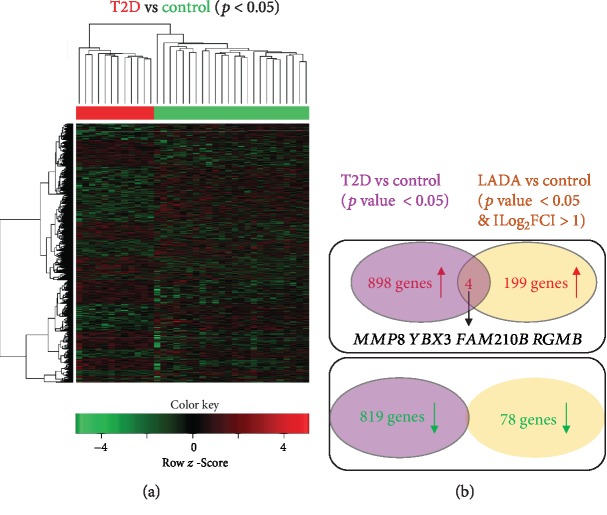
Overlapping analysis of DEGs between LADA and T2D. (a) The heat map shows the hierarchical clustering of the DEGs in T2D (solid red bar) and control (solid green bar) groups. Expression values in each row are *z*-score normalized. (b) Venn diagrams show the overlap of commonly upregulated or downregulated genes in T2D and LADA. The identified common genes are indicated.

To study the relationship between LADA and T1D/T2D, we compared the DEGs from our LADA dataset with those from T1D or T2D from the previous study (GSE9006) [10]. We identified 770 upregulated genes and 782 downregulated genes in T1D compared with healthy controls by analyzing the online dataset (Supplementary [Supplementary-material supplementary-material-1]). However, only 3 commonly upregulated genes (*MMP8*, *GPR146*, and *DNLZ*) and 1 commonly downregulated gene (*BNC2*) were identified between LADA and T1D datasets (Figure 5). Similarly, we identified 898 upregulated genes and 819 downregulated genes in T2D compared with healthy controls (Supplementary [Supplementary-material supplementary-material-1]). There were 4 commonly upregulated genes (*MMP8*, *YBX3*, *FAM210B*, and *RGMB*) but no commonly downregulated gene identified between LADA and T2D datasets (Figure 6).

## 4. Discussion

Here, we did a comparative analysis of the transcriptome profiles of LADA patients in Eastern China, followed by the ontology and KEGG pathway analyses showing these DEGs in LADA to be highly related to immune function and cell death and growth, respectively. In line with the previous findings [11], we also found that there are distinct transcriptome profiles between LADA and T1D and T2D.

In T1D, *β*-cell destruction is thought to be largely mediated by autoreactive T cells and autoantibody-induced autoimmune attack [12, 13]. The presence of autoimmune antibodies against *β*-cells is one of the diagnostic criteria of LADA, suggesting that the immune-mediated *β*-cell death also plays pivotal roles in the pathogenesis of LADA. In line with this, the top KEGG pathway classification analysis identified cell growth and death as the top pathway in DEGs, indicating that a large amount of DEGs were involved in the regulation of cell survival in LADA.

Like other forms of diabetes, the immune response is also critically involved in the pathogenesis of LADA. In line with this, our RNA-seq analysis revealed that the DEGs were enriched in GO terms of the immune response, such as chemokine activity, chemokine-mediated signaling, and neutrophil chemotaxis. For example, *CXCL8*, *CCL2*, and *CCL23* were confirmed to be upregulated in LADA. CXCL8 was originally identified as a potent neutrophil chemotactic factor by acting on G-protein-coupled receptors CXCR1 and CXCR2 [14, 15]. Emerging evidence suggests that neutrophils play important roles in the pathogenesis of diabetes, including LADA [16]. Gene products of CCL2 and CCL23 are ligands for chemokine receptors CCR1 and CCR2, respectively. Both CCL2 and CCL23 are potent chemoattractants for monocytes [17, 18]. Increased expression of neutrophil and monocyte chemoattractants may reflect the augmented inflammation in LADA since neutrophils act as the first-line-of-defense cells recruited to the site of inflammation [19]. Besides, monocytes and their macrophage progeny play important roles in the development of chronic inflammation [20]. The upregulation of chemokines and chemokine-mediated signaling supports the nature of inflammation in LADA.

Although there is the conventional perception that immune-mediated processes are not relevant to the pathogenesis of T2DM, low-grade inflammation that characterizes visceral obesity is linked to an autoimmune process that could influence the pathogenesis of T2DM [21]. Here, we found that LADA exhibited a dramatic increase of platelet activation-related genes, such as *PF4*, *PF4V1*, and *PPBP*. These data suggest that like other types of diabetes, LADA is also characterized by a chronic inflammatory response. Therefore, the heterogeneity of LADA may represent a progressive phenotypic spectrum between the two most common forms of diabetes mellitus, T1DM and T2DM.

We also noticed that our LADA patients either had insulin treatment or not (Supplementary [Supplementary-material supplementary-material-1]). We found the DEGs, which are identified between LADA and control patients (Supplementary [Supplementary-material supplementary-material-1]), showing the different expression patterns between LADA patients with or without insulin treatment (Supplementary Tables [Supplementary-material supplementary-material-1] and [Supplementary-material supplementary-material-1]). Expectedly, the top changed genes (*e.g.*, *SPATA6L*) between insulin treatment or non-insulin treatment LADA patients were associated with insulin response. For example, *SPATA6L* (Spermatogenesis Associated 6-Like Protein), upregulated in LADA compared to the control group (Supplementary [Supplementary-material supplementary-material-1]), had relatively higher expression in insulin treatment LADA patients than that in non-insulin treatment ones (Supplementary [Supplementary-material supplementary-material-1]). Thais et al. reported that *SPATA6* was dysregulated in the development of type 1 diabetes mellitus in nonobese diabetic mice [22] and Georg et al. recently found *SPATA6* to be one of the novel genes mediating *β*-cell failure [23]. Interestingly, these genes with similar changes (e.g., *LGALS3*, also named Galectin 3, Supplementary [Supplementary-material supplementary-material-1]) between insulin treatment or non-insulin treatment LADA patients seem to be regulated independently of insulin. Of note, Galectin 3 (*LGALS3*) has been reported to cause insulin resistance [24]. Therefore, these genes with similar changes between insulin treatment or non-insulin treatment LADA patients (Supplementary Tables [Supplementary-material supplementary-material-1] and [Supplementary-material supplementary-material-1]) may play important roles in insulin resistance.

There are some limitations to our study. First, although islet-infiltrating immune cells are presumably in equilibrium with circulating pools, they are diluted in the circulation. Therefore, our transcriptome analysis of sampled PBMCs may be improved by the analysis of fractionated PBMCs or pancreatic islets, particularly about the resolution in an understanding of the subset of cells. This idea was supported by the analysis of fractionated PBMCs (e.g., monocyte [25]). For example, *CCL2* and *EMP1* were identified in our DEGs of LADA to have a 3.3- and 2.46-fold increase, respectively (Supplementary [Supplementary-material supplementary-material-1]), while analysis of monocytes showed they had a 4.62- and 3.49-fold increase [25]. The differences about these fold changes of genes may lead to more findings, evidenced by CCL2 that was found to be a common DEG between LADA and T1D or T2D in the analysis of monocytes [25] though it is not a common gene in our analysis (Figures 5 and 6). Second, we identified these DEGs in LADA with the q-PCR validation of their gene expression. Further investigation of the regulatory mechanism of gene expression will aid in understanding how LADA develops. Particularly, epigenetic regulators can precisely turn off or on gene expression upon different stimulations. Third, more conclusions can be made by the recruitment of a large number of patients.

Taken together, our current study showed that the DEGs of LADA are highly involved in immune response and cell death and survival, resembling classical types of diabetes. On the other hand, few common DEGs between LADA and T1D/T2D make LADA a unique type of diabetes, requiring careful discrimination to reduce the rate of misdiagnosis and improve therapeutic treatment.

## Data Availability

All the raw data of RNA-seq were deposited into GEO databases (GSE136053).

## Supplementary Materials

Supplementary 1 Table S1: DEGs between LADA and healthy patients.

Supplementary 2 Table S2: top GO term analysis of DEGs between LADA and healthy patients.

Supplementary 3 Table S3: KEGG pathway classification analysis of DEGs between LADA and healthy patients.

Supplementary 4 Table S4: top KEGG pathway of DEGs between LADA and healthy patients.

Supplementary 5 Table S5: DEGs between TID and healthy patients.

Supplementary 6 Table S6: DEGs between T2D and healthy patients.

Supplementary 7 Table S7: LADA patient information.

Supplementary 8 Table S8: subgroup analysis of the upregulated DEGs between LADA and healthy patients based on insulin treatment or not.

Supplementary 9 Table S9: subgroup analysis of the downregulated DEGs between LADA and healthy patients based on insulin treatment or not.
